# Role of *MBL2* Polymorphisms in Sepsis and Survival: A Pilot Study and In Silico Analysis

**DOI:** 10.3390/diagnostics12020460

**Published:** 2022-02-11

**Authors:** Mohammed Y. Behairy, Ali A. Abdelrahman, Hoda Y. Abdallah, Emad El-Deen A. Ibrahim, Hany R. Hashem, Anwar A. Sayed, Marwa M. Azab

**Affiliations:** 1 Department of Microbiology and Immunology, Faculty of Pharmacy, University of Sadat City, Sadat City 32958, Egypt; mohamedyehya950@gmail.com; 2 Department of Microbiology and Immunology, Faculty of Pharmacy, Suez Canal University, Ismailia 41522, Egypt; dr_ali_abdellah@yahoo.com; 3 Medical Genetics Unit, Department of Histology and Cell Biology, Faculty of Medicine, Suez Canal University, Ismailia 41522, Egypt; hoda_ibrahim1@med.suez.edu.eg; 4 Center of Excellence in Molecular and Cellular Medicine, Faculty of Medicine, Suez Canal University, Ismailia 41522, Egypt; 5 Department of Anesthesia, Intensive Care and Pain Management, Faculty of Medicine, Suez Canal University, Ismailia 41522, Egypt; ee_ahmed@yahoo.com; 6 Department of Microbiology and Immunology, Faculty of Pharmacy, Fayoum University, Fayoum 63514, Egypt; hra11@fayoum.edu.eg; 7 Department of Medical Microbiology and Immunology, Taibah University, Madinah 42353, Saudi Arabia; 8 Department of Surgery and Cancer, Imperial College London, London SW7 2AZ, UK

**Keywords:** MBL, polymorphism, infection, sepsis, survival

## Abstract

Sepsis is a serious infection-induced syndrome with serious ramifications, especially in intensive care units. Global concern motivated the investigation of the role of related genes’ polymorphism in predicting the liability to infection, sepsis, septic shock and survival. Among these genes is the gene encoding mannose-binding lectin (MBL), with its remarkable importance in the immune system. However, the previous studies showed conflicting results and ambiguity that urged us to engage with this issue in the Egyptian population. Prediction of functional and structural impacts of single nucleotide polymorphisms (SNPs) was done using in silico methods. A prospective observational study was conducted in intensive care units; one hundred and thirty patients were followed up. Genotyping was performed using real-time polymerase chain reaction (RT-PCR) technology. MBL SNPs showed a remarkable high frequency in our population, as well. No significant association was found between *MBL2* genotypes and any of our analyses (sepsis, septic shock and survival). Only septic shock and age were independently associated with time of survival by Cox regression analysis. Our study may confirm the redundancy of MBL and the absence of significant impact on sepsis liability and mortality in adult patients.

## 1. Introduction

Sepsis is “a life-threatening organ dysfunction caused by a dysregulated host response to infection” [1]. This infection-induced syndrome is a major concern, especially in intensive care units, beyond its complicated manifestation, septic shock [2]. A recent global study estimated that, in 2017, the world witnessed 48.9 million cases of sepsis worldwide with 11 million sepsis-related deaths representing one fifth of all world deaths [3]. Moreover, the mortality of hospital-treated sepsis was estimated to be 26.7%, and the mortality of ICU-treated sepsis was estimated to reach 41.9% [4]. In addition, World Health Organization’s (WHO) seventieth assembly urged all WHO member states to improve the prevention, recognition and management of sepsis, considering it a global priority [5].

This global concern led to continuous efforts to search for robust diagnostic methods for the early prediction of sepsis, as early diagnosis is considered a priority for proper management of sepsis, leading to investigating the role of genetic polymorphisms in both the prediction of sepsis and its mortality rate, as well [6]. The believed role of genetic variants in sepsis pathogenesis and in individual sepsis susceptibility in addition to the importance of mannose-binding lectin (MBL) in the immune system attracted attention to its encoding gene, *MBL2,* and the possible roles of its variants in increasing liability to developing infection and sepsis [7,8].

MBL is a key player of the innate systemic protection against invading pathogens [9]. MBL is a pattern-recognition molecule that activates the complement system through the lectin pathway [10]. The MBL could identify a wide array of pathogens through carbohydrate moieties on their surfaces leading to complement activation in addition to further opsonization, phagocytosis enhancement and enhancement of the adaptive immune system [11,12]. The *MBL2* gene is located on chromosome 10 (q11.2–q21); this gene has three commonly studied polymorphisms on exon 1: rs1800450G/A, termed A/B in codon 54; rs1800451G/A, termed A/C in codon 57; and rs5030737C/T, termed A/D [13,14]. These three polymorphisms are called structural variants due to their modification of the subsequent protein structures and their role in preventing the assembly of oligomers of *MBL2* [14,15]. Moreover, mutations in codon 54 and codon 57 are found to be associated with dramatically low concentrations of mannose-binding protein, with its ramifications for immune function [16,17]. Meanwhile, MBL studies have found an astonishing high level of mutations in codon 54 and codon 57 in many populations, which lead to many hypotheses and much debate about the real role of these mutations and pushed for more investigation into their implications for infectious diseases [18,19]. Therefore, many studies investigated the association of exon 1 polymorphisms with the risk of infectious diseases and sepsis, and gave conflicting results [14,20,21,22,23]. These conflicted results indicated the need to further investigate, to identify whether single-nucleotide polymorphisms (SNPs) in this significantly important gene could be used for predicting a defect in our immunity towards infection and sepsis.

In this study, our aim was to investigate the potential role of codon 54 and codon 57 polymorphisms in the susceptibility to sepsis and septic shock in Egyptian population and in survival as well.

## 2. Materials and Methods

### 2.1. Ethics Statement

**Figure 1:**
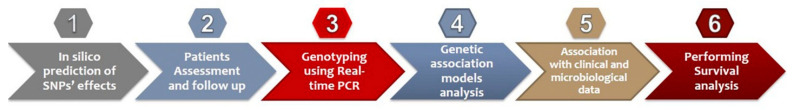
The specific objectives of the study work.

The protocol of this study was approved by the Research Ethics Committee at Suez Canal University with the reference No. (201911PHDH1). All subjects gave an informed consent or it was given by their next of kin. A chart illustrating the specific objectives of our work is shown in (Figure 1).

### 2.2. In Silico Analysis

#### 2.2.1. General Information

General information about *MBL2* gene was obtained from Ensembl and the National Center for Biotechnology Information (NCBI) databases. Gene ontology information was collected from Genecards.org, and we depended on compartments.jensenlab.org for subcellular localization data. The STRING biological database was used for analyzing predicted protein–protein interaction and gene co-expression. Ensembl and dbSNP were used for obtaining general information about our two SNPs: rs1800450 and rs1800451. We also depended on (https://web.expasy.org (last accessed on 17 August 2021)) for data related to the impact of these variants on protein sequences, for which UniProtKB/Swiss-Prot databases were used as the source of this information.

#### 2.2.2. Analyzing the Effect of Variants on Protein Function

The functional consequences of our two variants on protein function were predicted using five bioinformatics tools in order to strengthen the accuracy and efficacy of our analysis: 1—SIFT (sorting intolerant from tolerant) uses sequence homology, in addition to amino acids’ physical properties, to predict the impact of variants on protein function (https://sift.bii.a-star.edu.sg (last accessed on 17 August 2021)) [24]; 2—PolyPhen-2 (polymorphism phenotyping v2), depends on a comparative approach in additional to a physical one for predicting the impacts of variants (http://genet-ics.bwh.harvard.edu/pph2 (last accessed on 17 August 2021)) [25]; 3—PANTHER (protein analysis through evolutionary relationship) uses calculations of the evolutionary preservation of amino acids for predicting whether there is a likelihood that a nonsynonymous variant has functional consequences (http://www.pantherdb.org/tools/csnpScoreForm.jsp (last accessed on 21 September 2021)) [26]; 4—PROVEAN (protein variation effect analyzer) depends on blast hits for calculating delta alignment scores and eventually computing a PROVEAN score, with −2.5 being the cutoff (http://provean.jcvi.org/seq_submit.php (last accessed on 21 September 2021)) [27]; 5—SNPs and GO uses a protein’s functional annotation to predict the effects of SNPs (https://snps.biofold.org/snps-and-go/snps-and-go.html (last accessed on 21 September 2021)) [28].

#### 2.2.3. Identifying Variants’ Locations on MBL Protein Domains

InterPro tool was used to identify the SNPs locations on MBL protein conserved domains (https://www.ebi.ac.uk/interpro/ (last accessed on 21 September 2021)). InterPro is a bioinformatics tool used to analyze the function of protein and identify its functional sites and domains [29].

#### 2.2.4. Analyzing Variants Impact on Protein Stability

I-Mutant 2.0 was used for predicting the stabilities of MBL proteins from the rs1800450 and rs1800451 SNPs (https://folding.biofold.org/i-mutant/i-mutant2.0.html (last accessed on 21 September 2021)) [30]. I-Mutant 2.0 was tested effectively on the ProTherm database, considered the largest experimental database regarding protein mutations [31].

#### 2.2.5. Analysis of Evolutionary Conservation of MBL Protein Sequences

The ConSurf server was used for this analysis (https://consurf.tau.ac.il (last accessed on 13 September 2021)). This bioinformatics tool was used to analyze MBL protein sequences for evolutionarily conserved positions using phylogenetic relationships found in homologous sequences [32,33].

#### 2.2.6. Analyzing Structural Impacts of Variants

The HOPE tool was used to analyze the structural impacts of rs1800450 and rs1800451 on the MBL protein (https://www3.cmbi.umcn.nl/hope (last accessed on 13 September 2021)). The HOPE tool is a variant analysis server with which SNPs’ impacts on protein structures could be analyzed (Venselaar et al., 2010) [34].

### 2.3. Study Design

This is a prospective observational study that was conducted in intensive care units in Suez Canal university hospitals. All patients with a proven infection were included for a period of 7 months which was extended for another 5 months with the further inclusion of all admitted patients, including control patients without infection or sepsis in these last 5 months. The exclusion criteria were age less than 18 years, pregnancy, immunodeficiency and receiving radiation therapy or chemotherapy. All patients were followed during their hospital stay for developing infection, sepsis or septic shock and for their fate and survival. Routine cultures of blood, urine, sputum and pus were drawn to inspect infection and identify causative pathogens. Daily assessment and evaluation were performed to inspect the development of sepsis and septic shock according to “The Third International Consensus Definitions for Sepsis and Septic Shock (Sepsis-3)” [1].

In addition, upon admission a general examination was performed accompanied by measuring vital signs (heart rate, blood pressure, temperature respiratory rate and central venous pressure) and performing the needed laboratory investigations (complete blood count, creatinine, serum calcium, potassium, sodium, arterial blood gas). Additionally, calculating ICU score systems, such as Acute Physiology and Chronic Health Evaluation (APACHE II) score and sequential organ failure assessment (SOFA) score, was conducted as well.

### 2.4. Genotyping

DNA extraction from venous blood was performed using a QIAamp DNA Blood Mini kit (Cat. No. 51104, QIAGEN; Hilden, Germany). The purity and the concentration were checked using a NanoDrop ND-1000 (NanoDrop Tech., Inc. Wilmington, DE, USA). DNA samples were stored at −80 °C until further processing. SNPs of *MBL2*, both codon 54 (rs1800450G/A, termed A/B) and codon 57 (rs1800451G/A, termed A/C), were identified using the real-time polymerase chain reaction (RT-PCR) protocol with TaqMan Genotyping assays. The assay ID for rs1800451 was C___2336608_20 and the assay ID for rs1800450 was C___2336609_20. Reaction components were obtained from Applied Biosystems (Foster City, CA, USA). The PCR was performed in a reaction volume of 25 μL, including 12.5 μL TaqMan genotyping master mix, No AmpErase UNG (2×), 1.25 μL TaqMan SNP genotyping assay mix and 20 ng genomic DNA diluted with DNase-RNase free water to 11.25μL. After that, StepOne™ real time PCR system (Applied Biosystems, Foster City, CA, USA) was used for the amplification, under the following conditions: initial holding step of 95 °C for 10 min, then 40 cycles of 95 °C for 15 s and 60 °C for 1 min. The allelic discrimination depended on SDS software version 1.3.1 (Applied Biosystems). The steps were carried out blindly with regard to sepsis/culture/control groups. Additionally, re-genotyping of 20% of the samples was done on randomly selected samples with 100% concordance.

### 2.5. Statistical Analysis

Statistical Package for the Social Sciences (SPSS) for windows software, version 24 and Microsoft^®^ Excel 2010 was used for statistical analysis. Qualitative variables were expressed as frequency and percentage, the comparison between groups was performed by Chi-square (χ^2^) test or Fisher’s exact tests. Quantitative variables were expressed as mean ± standard deviation (SD), normally distributed quantitative variables were compared using student’s t test and one-way ANOVA tests, while non-normally distributed variables were compared using Mann–Whitney U test and Kruskal–Wallis test. A *p*-value less than 0.05 was considered statistically significant. Odds ratios (OR) were calculated with a 95% confidence interval (CI). Hardy–Weinberg equilibria (HWE) were calculated. Survival analysis was performed beginning with calculating Kaplan–Meier estimates of overall survival and performing log rank, Breslow, and Tarone–Ware tests to find Kaplan–Meier estimates for survival. Cox regression analysis was performed as well, to explore the independent associations with time of survival.

## 3. Results

### 3.1. In Silico Analysis

#### 3.1.1. General Information

**Figure 2:**
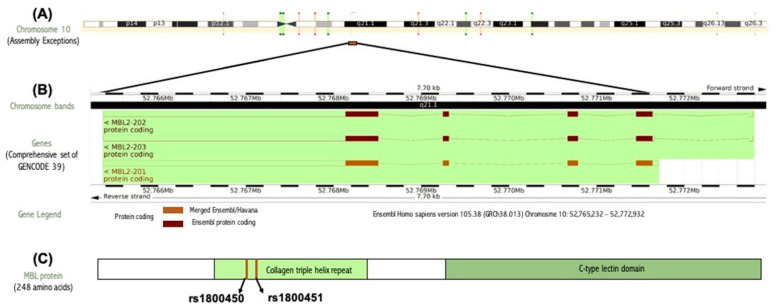
(**A**) Gene localization of the *MBL2* gene in the long arm of chromosome 10 generated by ensemble databases (http://ensembl.org/ (last accessed on 4 February 2022). (**B**) *MBL2* gene region in detail generated by ensemble databases (http://ensembl.org/ (last accessed on 4 February 2022). (**C**) MBL protein diagram representing its important domains and the positions of the SNPs under study.

**Figure 3:**
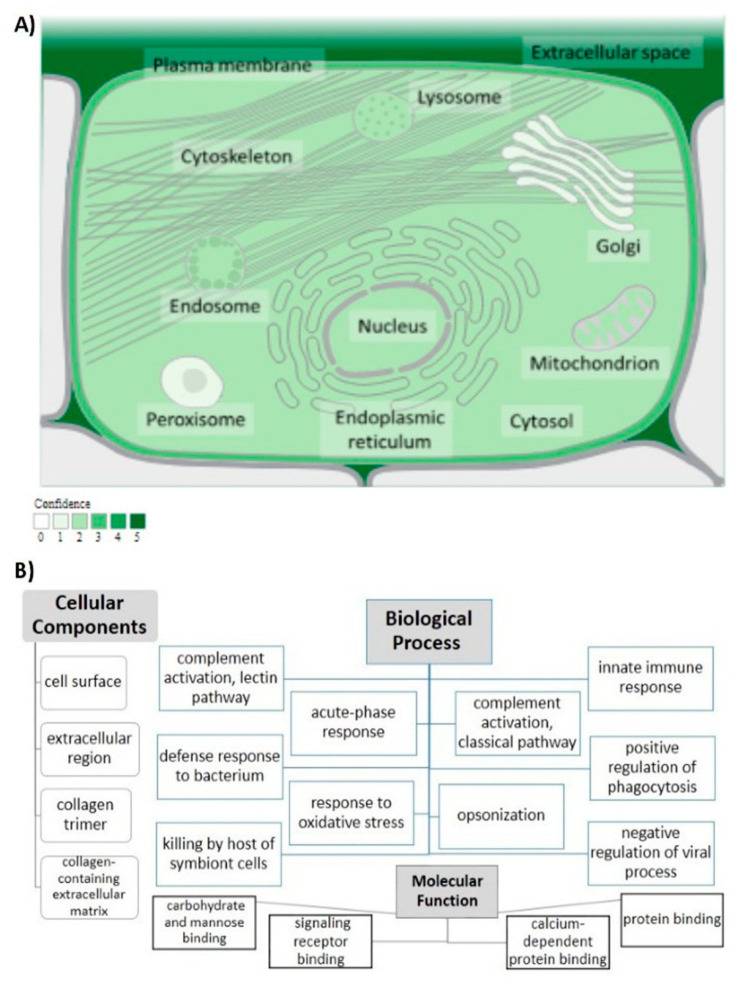
MBL protein functional analysis. (**A**) Subcellular localization of MBL protein. Confidence of association is color-coded with a gradient from light green, indicating low confidence, to dark green, for high confidence; from (https://www.genecards.org/ (last accessed on 4 February 2022)), with (https://compartments.jensenlab.org/ (last accessed on 4 February 2022)) as the source of the image. (**B**) Analysis of gene ontology. The cellular components, biological process and molecular function of MBL are illustrated.

**Figure 4:**
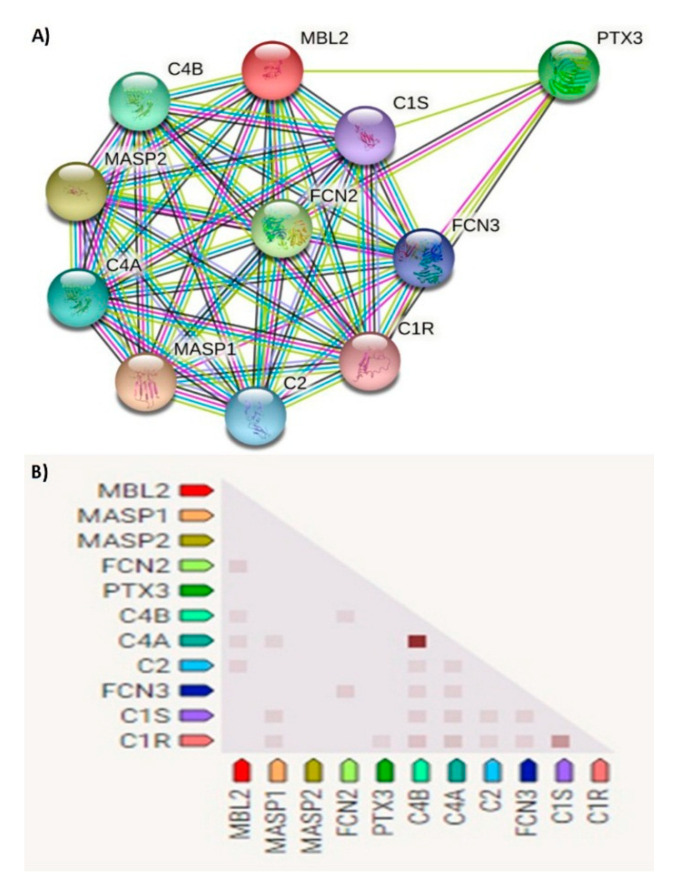
(**A**) Network of predicted protein–protein interactions of MBL protein. Analysis by STRING databases (version 11.5). The network shows the predicted summary of associations of a specific group of proteins. The nodes represent proteins, while the edges signify the predicted associations. The edges may have any of seven different colored lines, with different indications. Red lines—fusion evidence; blue lines—co-occurrence evidence; green lines—neighborhood evidence; light blue lines—database evidence; purple lines—experimental evidence; yellow lines—text-mining evidence; black lines—co-expression evidence. MASP1—mannan-binding lectin serine peptidase 1; MASP2—Mannan-binding lectin serine protease 2; FCN2—Ficolin-2; PTX3—pentraxin-related protein PTX3; C4B—complement C4-B; C4A—complement C4-A; C2—complement C2; FCN3—ficolin-3; C1S—complement C1s subcomponent; C1R—complement C1r subcomponent. (**B**) Gene co-expression matrix. In the triangular matrices, it is the intensity of color that indicates the level of confidence about the functional association between two proteins, given data about overall expression in the organism. Functional association is predicted by co-expression. *MBL2* shows co-expression with *FCN2, C4B, C4A* and *C2* with scores of 0.114, 0.099, 0.103 and 0.096, respectively (https://string-db.org (last accessed on 15 August 2021)).

The human *MBL2* gene (ENSG00000165471) is located on 10q21.1, the gene localization and the *MBL2* region in detail are illustrated in Figure 2A,B. It is a protein-coding gene with a length of 7405 nt., its location is at the Chromosome 10: 52,765,380–52,772,784 reverse strand according to the Genome Reference Consortium Human Build 38 patch release 13 (GRCh38.p13) and its NCBI reference sequence is (NC_000010.11). The gene has three transcripts (ensemble.org). The *MBL2* gene encodes mannose-binding lectin (also called mannose-binding protein C or mannan-binding protein), which belongs to the collectin family. Figure 2C illustrates a MBL protein diagram representing its important domains and the positions of the SNPs under study. Figure 3A illustrates the subcellular localization of MBL protein. It plays an important role in innate immunity by recognizing N-acetylglucosamine and mannose on microorganisms, such as bacteria, viruses and yeasts, and binds to them, leading to the activation of the complement system. (https://www.ncbi.nlm.nih.gov/gene?cmd=Re-trieve&dopt=Graphics&list_uids=4153# (last accessed on 17 August 2021)). The gene ontology (Figure 3B) shows that the biological process of our gene includes complement activation, opsonization and defense response to bacterium, while *MBL2* gene molecular functions comprise signaling receptor binding, protein binding, mannose and carbohydrate binding (https://www.genecards.org/cgi-bin/carddisp.pl?gene=MBL2 (last accessed on 16 August 2021)). Figure 4A illustrates the predicted interactions between MBL and other proteins that shows the existing interaction between MBL and many important proteins in the complement system and immune system that indicate the importance of MBL in the immune system, as the functional interactions of proteins represent a basis for completing biological functions [35]. In addition, the gene’s co-expression is illustrated in Figure 4B.

**Figure 5:**
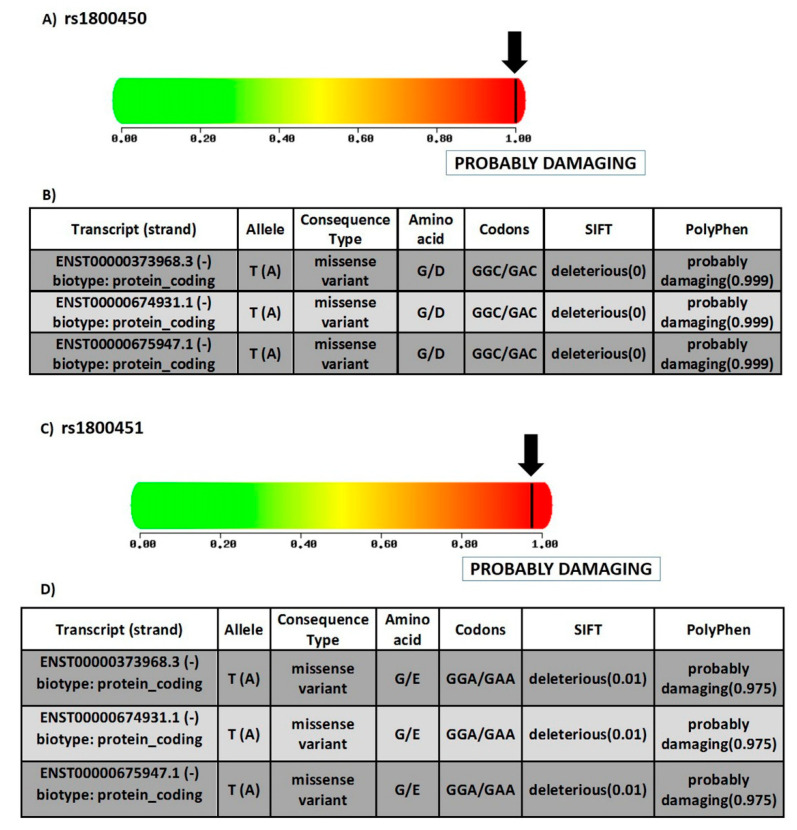
Structural and functional consequences of SNPs under study. (**A**) Prediction of the effect of rs1800450 on the function of human protein, depending on evolutionary and physical considerations, with a score varying from zero (benign) to one (damaging) (https://doi.org/10.1038/nmeth0410-248 (last accessed on 17 August 2021)). (**B**) Table displaying the different transcripts of rs1800450, alleles, consequence type, fate of amino acid, codons, SIFT score and PolyPhen score. G—glycine; D—aspartate (source of data: ensemble.org). (**C**) prediction of the effect of rs1800451 on the function of human protein depending on evolutionary and physical considerations, with a score varying from zero (benign) to one (damaging) (https://doi.org/10.1038/nmeth0410-248 (last accessed on 17 August 2021)). (**D**) Table displaying the different transcripts of rs1800451, alleles, consequence type, fate of amino acid, codons, SIFT score and PolyPhen score. G—glycine; E—glutamate (source of data: ensemble.org).

SNP rs1800450 is located at chromosome 10, position 52771475 (forward strand); it is also an exonic variant that comprises two alleles, C and T, where C is the ancestral allele. The minor allele frequency is 0.12 (T). This variant is a missense mutation that causes change of the amino acid glycine (G) to the amino acid aspartate (D) at position 54 (Figure 5A,B). Meanwhile, rs1800451 is located at chromosome 10 position 52771466 (forward strand), it is also an exonic variant with two alleles, C and T. The ancestral allele is C and the minor allele frequency equals 0.08 (T). It is also a missense mutation that causes the change of the amino acid glycine (G) to the amino acid glutamate (E) at position 57 (Figure 5C,D).

#### 3.1.2. Prediction of SNPs Impact on MBL Protein Function

For rs1800450, the SIFT tool revealed a score of 0.999. Thus, the SNP prediction showed a deleterious effect, which indicated that the substitution of the amino acid would lead to adverse effects on the function of the protein [24]. Moreover, the PolyPhen-2 tool revealed a score of 1.00 using the HumVar model, which designated the variant as a probably damaging mutation (Figure 5A). The Panther tool showed a probably damaging effect that the missense mutation may lead to a main role in causing human disease [26] with a probability of deleterious effect (Pdel) of 0.57. PROVEAN tool showed deleterious effect with a PROVEAN score of (−6.104). SNPs and GO indicated a prediction of a disease-associated SNP, with a reliability index of 7.

Meanwhile, for rs1800451, the SIFT tool revealed a score of 0.999. Consequently, the SNP prediction showed a deleterious effect, and an effect on protein function as well. PolyPhen-2 gave a score of 0.975 with the HumVar model, which designated the variant as a probably damaging mutation as well (Figure 5C). Panther also showed a probably damaging effect with rs1800451, with the probability of a deleterious effect (Pdel) of 0.57 as well. PROVEAN showed a deleterious effect, with PROVEAN score of −7.428. SNPs and GO predicted a disease-associated SNP with a reliability index of 6.

#### 3.1.3. Determining Variants’ Locations on Protein Domains

Using InterPro showed that MBL protein is composed of a C-type lectin-like domain and a collagen triple-helix repeat (collagen-like). Both rs1800450 and rs1800451 are located on a collagen triple-helix repeat (Interpro entry: IPR008160).

#### 3.1.4. Predicting MBL Protein Stability with rs1800450 and rs1800451 SNPs

The impact of our variants (rs1800450 and rs1800451) on MBL protein stability was analyzed by I-Mutant 2.0 in terms of reliability index value (RI) and free-energy change values (DDG). The rs1800450 was revealed to cause a decrease in MBL protein stability with a reliability index of 5 and free energy change values (DDG) of (−1.37) Kcal/mol. Meanwhile, the rs1800451 was revealed to cause an increase in MBL protein stability with a reliability index of 4 and a free-energy change value (DDG) of (0.32) Kcal/mol.

#### 3.1.5. Evolutionary Conservation Analysis

**Figure 6:**
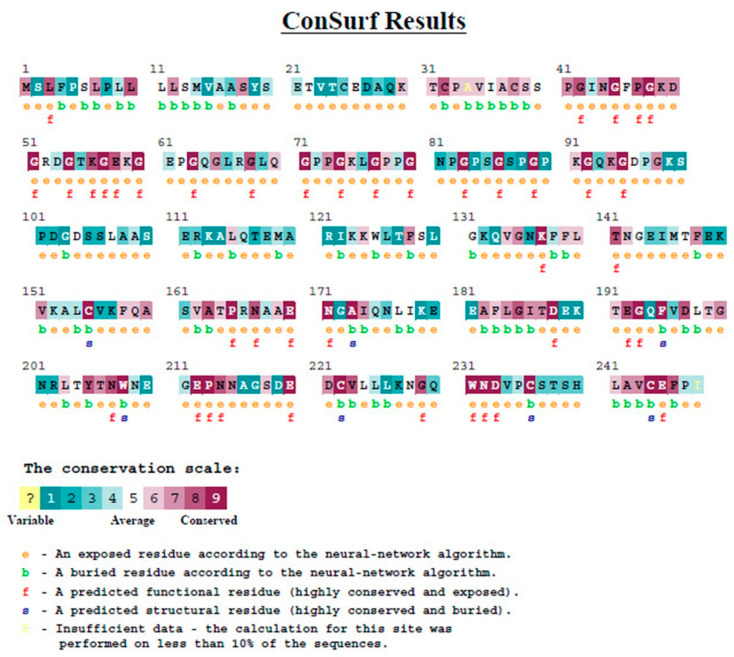
Evolutionary conservation analysis of MBL protein by Consurf.

Evolutionary conservation analysis of MBL protein was performed on the ConSurf server to estimate the degree of evolutionary conservation of MBL amino acids positions (Figure 6). The position 54 was revealed to be a functional residue, which is exposed and highly conserved. The position 57 was revealed also to be a functional residue, exposed and highly conserved as well.

#### 3.1.6. Analyzing Structural Effects of MBL Variants

Project HOPE was used to analyze structural effects of variants. With both SNPs, there was difference in size and charge between the new amino acid (aspartate and glutamic acid, for rs1800450 and rs1800451, respectively) and the original one, glycine. Unlike the neutral charge of glycine, both new amino acids had negative charges, which could cause repulsion of other negatively charged residues. Moreover, the bigger size of the new ones could cause bumps. Furthermore, the lost flexibility of glycine caused problems in this residue, with its unusual torsion angle that could cause disruption in structure. In addition, both aspartate and glutamic acid could disturb the collagen-like domain with their new properties, especially without the flexibility of glycine, which could be necessary to protein function.

### 3.2. Study Population

One-hundred-and-thirty patients were included in the study, of which 53 patients developed infection without developing sepsis, 38 patients developed sepsis and 39 patients did not develop infection or sepsis, composing the control group. The demographic and clinical features of ICU-admitted patients are summarized in (Table 1) according to their group. Some admission categories (neurology, infection, respiratory, trauma and gastrointestinal categories) and some variables, such as age, vital signs, Apache score and vascular concomitant disease, showed significant *p*-values. In addition, the frequencies of different causative microorganisms in the sepsis group and the infection group without sepsis were analyzed, as shown in (Table 2). However, no significant association was found between microorganisms’ frequency and the development of sepsis. Moreover, no significant value was found, with an odds ratio and 95% confidence interval as well (Table 2).

### 3.3. Genotype Analysis

Genotype frequencies, allele frequencies and carriage rate are mentioned in detail in (Table 3) with the odds ratio and confidence interval calculated. The frequency of the AA, AC and CC genotypes of rs1800451 were 109, 19 and 2, respectively, in accordance with Hardy–Weinberg equilibrium. While the genotypes of rs1800450 frequency were 90, 40 and 0 for AB, AA and BB, respectively, which gave significant value with Hardy–Weinberg equilibrium. Rs1800451 and rs1800450 did not show significant relationships with developing sepsis. In addition, all genetic association models were investigated with risk of sepsis (Table 4), but no significant association was observed.

### 3.4. Polymorphisms and Clinical Characteristics

Moreover, all clinical and laboratory variables were investigated for their relationships with rs1800451 or rs1800450 (Table 5). There was no association between our SNPs and developing septic shock or length of stay. The respiratory category of admission was found to have a significant association with rs1800450 (*p* = 0.011).

### 3.5. Survival Analysis

**Figure 7:**
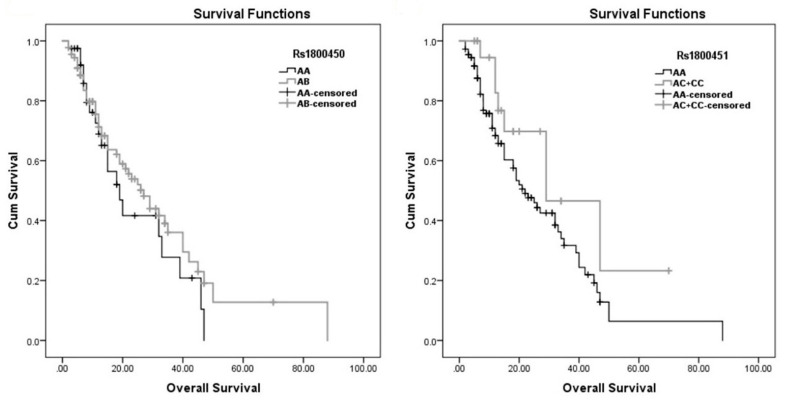
Kaplan–Meier estimates of overall survival.

Survival analysis was performed in a multistep approach in our study. First, Kaplan–Meier survival plots were created for the two SNPs (Figure 7). Then, we applied the log rank test, Breslow test and Tarone–Ware test, investigating the associations between all variables, including our two SNPs, and with survival (Table 6). This analysis resulted in a significant association with length of stay *p*-value = 0.000, 0.000 and 0.000, respectively. Another significant association was with infection category of admission, *p*-value = 0.018, 0.011 and 0.014, respectively, and with the neurology category of admission *p*-value = 0.030, 0.004 and 0.007, respectively. The chronic lung disease also showed significance with the log rank test and Tarone–Ware test, with *p*-value = 0.026 and 0.044, respectively. In addition, the number of infections gave a significant result with the Tarone–Ware test, giving a *p*-value = 0.048.

Furthermore, performing Cox regression analysis (Table 7) resulted in an independent association between septic shock and between time of survival, with a hazard risk of 2.882, confidence interval = 1.130–7.347 and *p*-value = 0.027. Additionally, an independent association of age with time of survival was found as well, with a hazard risk of 1.018, confidence interval = 1.004–1.034 and *p*-value = 0.015.

## 4. Discussion

The revealed contribution of polymorphisms in immune system genes to the liability and outcome of sepsis patients [6,36], in addition to the remarkable importance of MBL in the immune system motivated us to investigate this possible association between MBL genes variants and the liability to and the outcomes of this serious infectious disease.

All used bioinformatics tools showed damaging and deleterious impacts of MBL SNPs on MBL protein and that the SNPs have adverse effects on the function of our protein. Rs1800450 SNP was showed to decrease the stability of MBL protein. Furthermore, evolutionary conservation analysis showed that MBL SNPs were positioned at functional, exposed and highly conserved residues. In addition, the amino acid exchanges were predicted to cause disruption in protein structure with both variants.

The genotype frequencies of the SNPs in our study were in agreement with previous published data among the Egyptian population; the genotype frequency of rs1800451 was similar to the frequency found by Badawy and colleagues in Egyptian population [23], as well as those from studies in other populations [14,37]. The genotype frequency results of rs1800450 were very similar to that found by Nasr et al. in a recent study in an Egyptian population, with frequencies of 41.7%, 58.3%, 0.0% for the AA, AB and BB genotypes, respectively [38], similar to the results found by Badawy and colleagues [23]. The notable high frequency associated with these variants was observed previously, and attracted much attention, leading to many different hypotheses and an resolved debate concerning the real roles of these variants [18,19,39,40]. One hypothesis referred this accumulation of variants to a protective function, due to low MBL production associated with these genotypes, thus suggesting protection from host damage caused by excessive amount of inflammatory mediators [39,41,42], or suggesting protection against some intracellular organisms, such as Leishmania species, a serious intracellular parasite [43]. On the other hand, the advocates of another hypothesis excluded any selective pressure and found no statistical evidence of such pressure [44,45].

In our study, we found no relationship between our polymorphisms and sepsis susceptibility or susceptibility to infection, as there was no statistical difference between the three study groups according to genotype frequency, allele frequency or carriage rate. We found also no association between our SNPs and between developing septic shock. In addition, we did not find any statistical difference concerning risk by calculating odds ratios between the sepsis group, infection group and control group, according to the examined SNPs. The debates and conflicting results extended to previous studies regarding the role of MBL polymorphism in the susceptibility to infection, sepsis, septic shock and sepsis outcome, Gordon et al. found significant a relationship between exon 1 polymorphisms and between developing sepsis in adult an population in England [46]; Liu et al. also found a significant association between codon 54 polymorphism and the risk of sepsis in an adult Han Chinese population [8]. A South Korean study found an influence of codon 54 polymorphism on sepsis severity and developing septic shock but did not find any independent association between exon 1 polymorphisms and between sepsis incidence [47]. On the other hand, many studies were consistent with our results; a large study that was performed on more than 9000 adult Danish participants found no association between exon 1 polymorphisms and the risk of infectious diseases [48]. Moreover, Zhang and colleagues, in their meta-analysis, found no statistical significance between exon 1 polymorphisms and between sepsis susceptibility in adults [20]. In addition, a large cohort study performed on a European population found no significant association between our SNPs and other *MBL2* SNPs and between pneumococcal sepsis and community-acquired pneumonia (CAP) sepsis [21]. Our agreement with such large studies strengthened to our results. Moreover, a prospective Dutch study found no association between *MBL2* genotypes and the susceptibility to CAP and that *MBL2* genotypes could not be considered as factors with major risk for developing infection with CAP [49]. In addition, regarding neonatal sepsis in an Egyptian population, although an Egyptian cross sectional study found a prevalence of mutant allele B with neonatal sepsis that might indicate a possible role of allele B of codon 54 in neonatal sepsis [50], another case-controlled study with a larger sample size was conducted on neonates in an Egyptian population and found no association of *MBL2* SNPs with sepsis risk [23].

We did not find significant statistical association between our SNPs and between survival by using Log rank test, Breslow test and Tarone–Ware tests. Cox regression analyses did not reveal significance between our SNPs and survival as well. These results regarding sepsis survival are consistent with a large study of Mills and colleagues, who found no association between MBL variants and sepsis survival in adults [21] and with a large study on an adult Danish population, which concluded that MBL polymorphisms could not be considered as a major risk for mortality [48]. In addition, both Huh et al. and Gordon et al. found no influence of MBL variants on sepsis mortality [46,47]. Yet some inconsistent results could be noticed, as well; Garnacho-Montero and colleagues reported that *MBL2* polymorphisms were associated with mortality with pneumococcal sepsis in adult patients in Spain [51].

This absence of association of MBL variants with infection, sepsis and sepsis mortality could be explained by the redundancy of MBL [52,53]. This was supported by previous results—that other mechanisms could replace MBL function in the immune system [54]. The reason for the inconsistent findings, according to Mills and colleagues, was the underpowered studies conducted by most researchers [21]. This explanation agreed with our bioinformatics analysis about the deleterious effects of our SNPs on protein function and also with previous studies [16,17], as they referred the non-significant association in spite of these deleterious effects to the role of replacing immune mechanisms and the redundancy of MBL.

The other explanation is that the different ethnicities and geographical regions in these studies could be responsible for this inconsistency [20]. The susceptibility to infectious diseases and sepsis and their outcome represent a complex process that is determined by a combination of host genetic, environmental as well as pathogen factors [55,56]. Additionally, these genetic factors consist of frequent genes rather than one single gene [6]. Therefore, different ethnicities and geographical regions lead to different environmental factors and different frequencies of polymorphisms, which could be responsible for these conflicting results in different ethnicities and geographical regions.

Rs1800450 was found to have a significant association with the respiratory category of admission, this category consisted mainly of respiratory failure, chronic obstructive pulmonary disease (COPD) and pulmonary embolism patients. This group and its subgroups were too small for further analysis, but the previous data linking MBL variants with disease severity in noninfectious respiratory diseases [57,58] may indicate the need for further investigation in this point.

Survival analysis showed that number of infections in patient and infection category of admission were associated with survival time which confirmed previous results about the role of infection in increasing mortality risk in ICU [59,60]. The length of ICU stay was also significantly associated with survival time, in agreement with previous studies which showed increasing rate of mortality with the increase in length of ICU stay [61,62]. In addition, analysis confirmed association of septic shock with survival time agreed with previous studies [1,63] and performing Cox regression analysis found that only septic shock and age factors had independent association with time of survival. Finally, there were two limitations in the current study. First, the relatively small sample size, thus, large multi-center studies are recommended to confirm these results. Second, having analyzed only two polymorphisms in the *MBL2* gene.

## 5. Conclusions

In conclusion, MBL variants were found with high frequency in our population, agreeing with previous studies in the still unresolved debates about the nature of these high-frequency variants. No roles of the MBL variants (rs1800450, rs1800451) were found in the susceptibility to infection or in developing sepsis and septic shock. They also have no role in patients’ survival. The redundancy of MBL and presence of other compensatory immune mechanisms could be confirmed with our results; otherwise, the roles of different ethnicities and geographical regions could be responsible for the conflicting results.

## Figures and Tables

**Table 1:** Demographic and clinical features of ICU-admitted patients (control, infection without sepsis, sepsis groups) (*n* = 130).

Variables		All	Control	Infection without Sepsis	Sepsis	*p*-Value
Demographic Characteristics						
Number		130	39	53	38	
Age, years	median (IQR)	60.0 (22.3)	55.0 (29.0)	59.0 (33.5)	65.0 (17.3)	**0.005**
	≤40 years	28 (21.5%)	12 (30.8%)	14 (26.4%)	2 (5.3%)	
	≤60 years	40 (30.8%)	14 (35.9%)	15 (28.3%)	11 (28.9%)	**0.020**
	>60 years	62 (47.7%)	13 (33.3%)	24 (45.3%)	25 (65.8%)	
Sex	Male	76 (58.5%)	24 (61.5%)	33 (62.3%)	19 (50.0%)	0.45
	Female	54 (41.5%)	15 (38.5%)	20 (37.7%)	19 (50.0%)	
Vital signs	HR	90.0 (17.0)	90.0 (00.0)	100.0 (24.0)	90.0 (20.8)	**0.008**
	MAP	83.0 (14.0)	83.0 (00.0)	83.0 (24.5)	75.0 (20.0)	**0.047**
**Concomitant diseases**						
Diabetes	positive	45 (34.6%)	10 (25.6%)	17 (32.1%)	18 (47.4%)	0.12
Hypertension	positive	65 (50.0%)	16 (41.0%)	28 (52.8%)	21 (55.3%)	0.40
Vascular disease	positive	34 (26.2%)	4 (10.3%)	17 (32.1%)	13 (34.2%)	**0.025**
Chronic lung disease	positive	8 (6.2%)	2 (5.1%)	5 (9.4%)	1 (2.6%)	0.54
Chronic liver disease	positive	10 (7.7%)	2 (5.1%)	3 (5.7%)	5 (13.2%)	0.42
Chronic renal disease	positive	25 (19.2%)	5 (12.8%)	11 (20.8%)	9 (23.7%)	0.45
**ICU assessment**						
APACHE score	median (IQR)	15.0 (7.3)	12.0 (7.0)	16.0 (7.0)	16.5 (6.8)	**0.001**
Glasgow scale	median (IQR)	11.5 (8.0)	14.0 (9.0)	9.0 (7.5)	14.0 (8.0)	0.057
Length of stay, days	median (IQR)	13.0 (19.3)	10.0 (16.0)	15.0 (18.5)	13.5 (25.8)	0.386
Consequence	discharge	54 (41.5%)	19 (48.7%)	21 (39.6%)	14 (36.8%)	
	transferred	6 (4.6%)	1 (2.6%)	3 (5.7%)	2 (5.3%)	0.82
	death	70 (53.9%)	19 (48.7%)	29 (54.7%)	22 (57.9%)	
OS, days	median (IQR)	13.0 (20.5)	11.0 (15.0)	15.0 (20.0)	16.5 (28.3)	0.424
**Admission category**						
Renal	positive	4 (3.1%)	1 (2.6%)	1 (1.9%)	2 (5.3%)	0.69
Cardiovascular	positive	5 (3.8%)	2 (5.1%)	2 (3.8%)	1 (2.6%)	1.00
Infection	positive	24 (18.5%)	0 (0.0%)	8 (15.1%)	16 (42.1%)	**0.000**
Neurology	positive	36 (27.7%)	13 (33.3%)	19 (35.8%)	4 (10.5%)	**0.019**
Post-surgical	positive	19 (14.6%)	4 (10.3%)	6 (11.3%)	9 (23.7%)	0.17
Respiratory	positive	14 (10.8%)	2 (5.1%)	11 (20.8%)	1 (2.6%)	**0.013**
Trauma	positive	10 (7.7%)	7 (17.9%)	3 (5.7%)	0 (0.0%)	**0.009**
Other causes	positive	9 (6.9%)	4 (10.3%)	2 (3.8%)	3 (7.9%)	0.52
Gastrointestinal	positive	9 (6.9%)	6 (15.4%)	1 (1.9%)	2 (5.3%)	**0.042**

Data is shown as number (percentage) or median (IQR) for non-parametric statistics. IQR—interquartile range; HR—heart rate, in beats per minute; MAP—mean arterial pressure, in mmHg; OS—overall survival. Chi square (χ^2^) or Fisher’s exact tests were used for qualitative variables and the Kruskal–Wallis test was used for non-normally distributed quantitative attributes. Bold values are statistically significant at *p*-value < 0.05.

**Table 2:** Analysis of causative organisms in sepsis group and infection group without sepsis (*n* = 91).

Causative Organism	All	Infection without Sepsis	Sepsis	*p*-Value	Odds Ratio (95% CI)
*Enterobacter* spp.	10 (11.0%)	4 (7.5%)	6 (15.8%)	0.31	2.30 (0.60–8.78)
*Acinetobacter* spp.	11 (12.1%)	7 (13.2%)	4 (10.5%)	0.76	0.77 (0.21–2.85)
*Candida* spp.	3 (3.3%)	1 (1.9%)	2 (5.3%)	0.57	2.89 (0.25–33.07)
*Escherichia coli*	16 (17.6%)	11 (20.8%)	5 (13.2%)	0.35	0.58 (0.18–1.83)
*Gram negative bacilli*	9 (9.9%)	5 (9.4%)	4 (10.5%)	1.00	1.13 (0.28–4.52)
*Klebsiella* spp.	21 (23.1%)	13 (24.5%)	8 (21.1%)	0.70	0.82 (0.30–2.23)
*Pseudomonas* spp.	13 (14.3%)	6 (11.3%)	7 (18.4%)	0.34	1.77 (0.54–5.76)
*Staph* spp.	18 (19.8%)	12 (22.6%)	6 (15.8%)	0.42	0.64 (0.22–1.89)
*Streptococcus* spp.	4 (4.4%)	3 (5.7%)	1 (2.6%)	0.64	0.45 (0.05–4.51)
*Aeromonas* spp.	1 (1.1%)	1 (1.9%)	0 (0.0%)	1.00	0.45 (0.02 to 11.46)
*Proteus* spp.	2 (2.2%)	1 (1.9%)	1 (2.6%)	1.00	1.41 (0.09–23.20)
*Citrobacter* spp.	1 (1.1%)	1 (1.9%)	0 (0.0%)	1.00	0.45 (0.02 to 11.46)
*Serratia* spp.	1 (1.1%)	1 (1.9%)	0 (0.0%)	1.00	0.45 (0.02 to 11.46)

Data is shown as number (percentage). Chi square (χ^2^) or Fisher’s exact tests were used for qualitative variables. Statistical analysis at *p*-value <0.05.

**Table 3:** Genotype frequencies, allele frequencies and carriage rate with risk of sepsis.

	All	Control	Infection without Sepsis	Sepsis	*p*-Value	OR (95% CI)		
						Sepsis Group against Control Group	Sepsis Group against Infection Group	Infection Group against Control Group
Genotype Frequencies								
Rs1800451								
A/A	109 (83.8%)	32 (82.1%)	45 (84.9%)	32 (84.2%)	0.91	Reference		
A/C	19 (14.6%)	7 (17.9%)	7 (13.2%)	5 (13.2%)		0.71 (0.21 to 2.49)	1.00 (0.29 to 3.45)	0.71 (0.23 to 2.23)
C/C	2 (1.6%)	0 (0.0%)	1 (1.9%)	1 (2.6%)		3.00 (0.12 to 76.40)	1.41 (0.08 to 23.33)	2.14 (0.09 to 54.29)
P _HWE_	0.90	1.00	1.00	1.00				
**Rs1800450**								
A/A	40 (30.8%)	11 (28.2%)	15 (28.3%)	14 (36.8%)	0.63	Reference		
A/B	90 (69.2%)	28 (71.8%)	38 (71.7%)	24 (63.2%)		0.68 (0.28 to 1.65)	0.67 (0.26 to 1.76)	1.00 (0.40 to 2.49)
B/B	0	0	0	0		0.79 (0.01 to 43.12)	1.07 (0.02 to 57.49)	0.74 (0.01 to 40.25)
P _HWE_	**0.000**	**0.012**	**0.002**	**0.039**				
**Allele frequencies**								
**Rs1800451**								
A	237 (91.15%)	71 (91.0%)	97 (91.5%)	69 (90.8%)	0.98	Reference		
C	23 (8.85%)	7 (9.0%)	9 (8.5%)	7 (9.2%)		1.03 (0.34 to 3.09)	1.09 (0.39 to 3.08)	0.94 (0.33 to 2.64)
**Rs1800450**								
A	170 (65.4%)	50 (64.1%)	68 (64.2%)	52 (68.4%)	0.80	Reference		
B	90 (34.6%)	28 (35.9%)	38 (35.8%)	24 (31.6%)		0.82 (0.42 to 1.61)	0.83 (0.44 to 1.54)	1.00 (0.54 to 1.84)
**Carriage rate**								
**Rs1800451**								
A	128 (98.5%)	39 (100%)	52 (98.1%)	37 (97.4%)	0.07	Reference		
C	21 (16.2%)	7 (17.9%)	8 (15.1%)	6 (15.8%)		0.90 (0.28 to 2.94)	1.05 (0.34 to 3.29)	0.86 (0.29 to 2.56)
**Rs1800450**								
A	130 (100%)	39 (100%)	53 (100%)	38 (100%)	0.17	Reference		
B	90 (69.2%)	28 (71.8%)	38 (71.7%)	24 (63.2)		0.88 (0.43 to 1.78)	0.88 (0.46 to 1.70)	1.00 (0.53 to 1.89)

Values are shown as number (percentage). Chi square (χ^2^) or Fisher’s exact tests were used. OR (95% CI), odds ratio and confidence interval. Statistical analysis at *p* value < 0.05.

**Table 4:** Genetic association models for sepsis risk assessment.

	Genotype	Control	Infection without Sepsis	Sepsis	*p*-Value	OR (95% CI)		
						Sepsis Group against Control Group	Sepsis Group against Infection Group	Infection Group against Control Group
**rs1800451**								
Codominant	A/A	32 (82.1%)	45 (84.9%)	32 (84.2%)	0.91	Reference		
	A/C	7 (17.9%)	7 (13.2%)	5 (13.2%)		0.71 (0.21 to 2.49)	1.00 (0.29 to 3.45)	0.71 (0.23 to 2.23)
	C/C	0 (0.0%)	1 (1.9%)	1 (2.6%)		3.00 (0.12 to 76.40)	1.41 (0.08 to 23.33)	2.14 (0.09 to 54.29)
Dominant	A/A	32 (82.1%)	45 (84.9%)	32 (84.2%)	0.93	Reference		
	A/C-C/C	7 (17.9%)	8 (15.1%)	6 (15.8%)		0.86 (0.26 to 2.83)	1.05 (0.33 to 3.34)	0.81 (0.27 to 2.47)
Recessive	A/C-A/A	39 (100%)	52 (98.1%)	37 (97.4%)	0.75	Reference		
	C/C	0 (0.0%)	1 (1.9%)	1 (2.6%)		3.16 (0.12 to 80.02)	1.41 (0.09 to 23.20)	2.26 (0.09 to 56.90)
Over-dominant	A/A-C/C	32 (82.1%)	46 (86.8%)	33 (86.8%)	0.78	Reference		
	A/C	7 (17.9%)	7 (13.2%)	5 (13.2%)		0.69 (0.20 to 2.41)	1.00 (0.29 to 3.41)	0.70 (0.22 to 2.18)
**rs1800450**								
Codominant	A/A	11 (28.2%)	15 (28.3%)	14 (36.8%)	0.63	Reference		
	A/B	28 (71.8%)	38 (71.7%)	24 (63.2%)		0.68 (0.28 to 1.65)	0.67 (0.26 to 1.76)	1.00 (0.40 to 2.49)
	B/B	0	0	0		0.79 (0.01 to 43.12)	1.07 (0.02 to 57.49)	0.74 (0.01 to 40.25)
Dominant	A/A	11 (28.2%)	15 (28.3%)	14 (36.8%)	0.63	Reference		
	A/B-B/B	28 (0.0%)	38 (0.0%)	24 (0.0%)		0.67 (0.26 to 1.76)	0.68 (0.28 to 1.65)	1.00 (0.40 to 2.49)
Recessive	A/A-A/B	39 (100%)	53 (100%)	38 (100%)	1.00	Reference		
	B/B	0	0	0		1.03 (0.02 to 53.02)	1.39 (0.03 to 71.58)	0.74 (0.01 to 38.02)
Over-dominant	A/A-B/B	11 (28.2%)	15 (28.3%)	14 (36.8%)	0.63	Reference		
	A/B	28 (0.0%)	38 (0.0%)	24 (0.0%)		0.67 (0.26 to 1.76)	0.68 (0.28 to 1.65)	1.00 (0.40 to 2.49)

Values are shown as number (percentage). Chi square (χ^2^) or Fisher’s exact tests were used. OR (95% CI), odds ratio and confidence interval. Statistical analysis at *p* value < 0.05.

**Table 5:** Analysis for association of variants with clinical and laboratory characteristics.

**Variables**		**Codon 54 (rs1800450)**	**Codon 57 (rs1800451)**
		***p*** **-Value**	***p*** **-Value**
Demographic	Age, years	0.81	0.61
	Sex	0.88	0.90
Vital signs	HR, beats/min	0.36	0.47
	MAP, mm Hg	0.81	0.45
	SBP, mm Hg	0.42	0.49
	DBP, mm Hg	0.71	0.39
Concomitant diseases	Diabetes	0.65	0.44
	Hypertension	0.70	1.00
	Vascular disease	0.27	0.44
	Chronic lung disease	0.25	1.00
	Chronic liver disease	1.00	1.00
	Chronic renal disease	0.53	1.00
ICU assessment	APACHE score	0.75	0.80
	Glasgow scale	0.10	0.50
	Length of stay	0.22	0.24
	Sepsis	0.63	0.91
	Septic shock	0.78	0.76
	Death	0.84	0.17
	Overall survival	0.97	0.40
Admission category (cause of admission)	Renal	0.59	1.00
	Cardiovascular	1.00	0.59
	Infection	0.24	0.84
	Neurology	0.19	0.69
	Post-surgical	0.54	0.80
	Respiratory	0.011	0.38
	Trauma	0.72	1.00
	Other causes	1.00	0.11
	Gastrointestinal	1.00	0.67
Laboratory results	WBC, ×10^3^ cells/μL	0.46	0.58
	HB, g%	0.35	0.83
	Creatinine, mg/dL	0.55	0.74
**Variables**		**Codon 54 (rs1800450)**	**Codon 57 (rs1800451)**
		***p*** **-Value**	***p*** **-Value**
Causative organism	*Enterobacter* spp.	0.50	1.00
	*Acinetobacter* spp.	0.74	1.00
	*Candida* spp.	1.00	0.41
	*E. coli*	1.00	0.43
	*Gram-negative bacilli*	1.00	1.00
	*Klebsiella* spp.	0.81	0.17
	*Pseudomonas* spp.	1.00	0.75
	*Staph* spp.	0.42	0.30
	*Streptococcus* spp.	0.59	0.07
	*Aeromonas* spp.	1.00	1.00
	*Proteus* spp.	0.52	1.00
	*Citrobacter* spp.	1.00	1.00
	*Serratia* spp.	1.00	1.00
Type of culture	Blood	0.40	0.18
	Sputum	0.70	0.29
	Urine	0.54	1.00
	Pus	0.19	1.00
	CSF	0.31	1.00
No. of infections		0.48	0.31

CSF—Cerebrospinal Fluid; DBP—Diastolic Blood Pressure; HB—Hemoglobin; HR—Heart Rate; MAP—Mean Arterial Pressure; SBP—Systolic Arterial Pressure; WBC—White Blood Cell.

**Table 6:** Survival analysis in ICU-admitted patients.

Variables		Overall Comparisons		
		Log Rank	Breslow	Tarone–Ware
Demographic data	Age	0.154	0.247	0.180
	Sex	0.701	0.542	0.582
Concomitant disease	Diabetes	0.820	0.401	0.543
	Hypertension	0.536	0.377	0.418
	Vascular disease	0.141	0.361	0.271
	Chronic liver disease	0.891	0.979	0.891
	Chronic lung disease	**0.026**	0.064	**0.044**
	Chronic renal disease	0.124	0.126	0.122
ICU assessment	APACHE score	0.308	0.261	0.256
	Glasgow scale	0.115	0.228	0.175
	Length of stay	**0.000**	**0.000**	**0.000**
	Sepsis	0.807	0.797	0.793
	Septic shock	0.090	**0.020**	**0.038**
Admission category	Renal	0.571	0.396	0.442
	Cardiovascular	0.954	0.687	0.766
	Infection	**0.018**	**0.011**	**0.014**
	Neurology	**0.030**	**0.004**	**0.007**
	Post-surgical	0.273	0.279	0.264
	Respiratory	0.454	0.865	0.672
	Trauma	0.111	0.200	0.155
	Other causes	0.308	0.357	0.314
	Gastrointestinal	0.078	0.240	0.128
Laboratory results	WBC, x10^3^ cells/μl	0.165	0.062	0.080
	HB, g%	0.066	0.218	0.140
	Creatinine, mg/dL	0.064	0.144	0.117
	No. of infections	0.149	0.050	**0.048**
Molecular analysis	RS1800450	0.336	0.728	0.548
	RS1800451	0.116	0.093	0.102

Survival time is shown as mean and standard error. Log rank, Breslow and Tarone–Ware tests were used to find Kaplan–Meier estimates for survival. Bold values are statistically significant at *p* < 0.05. Quantitative variables were categorized by their medians. HB—Hemoglobin; WBC—White Blood Cell.

**Table 7:** Survival analysis in ICU admitted patients.

Variables		HR	95% CI	*p*-Value
Demographic data	Age	1.018	1.004–1.034	0.015
	Sex	1.262	0.753–2.117	0.377
ICU assessment	APACHE core	1.003	0.970–1.036	0.866
	Glasgow scale	1.056	0.988–1.129	0.107
	Septic shock	2.882	1.130–7.347	0.027
	Sepsis (sepsis–no sepsis)	0.455	0.191–1.084	0.075
	No. of infections	0.738	0.541–1.006	0.055
Molecular analysis	RS1800451 (AA, AC + CC)	1.599	0.742–3.444	0.231
	RS1800450 (AA, AB + BB)	1.108	0.632–1.940	0.720

HR—hazard risk; CI—confidence interval. Cox proportional hazard regression analysis was performed.

## Data Availability

All supporting data of the study are available from the corresponding authors upon request.
